# Testing a Combination of Markers of Systemic Redox Status as a Possible Tool for the Diagnosis of Late Onset Alzheimer's Disease

**DOI:** 10.1155/2018/2576026

**Published:** 2018-09-09

**Authors:** Giovanni Zuliani, Angelina Passaro, Cristina Bosi, Juana Maria Sanz, Alessandro Trentini, Carlo M. Bergamini, Davide Seripa, Antonio Greco, Monica Squerzanti, Roberta Rizzo, Giuseppe Valacchi, Carlo Cervellati

**Affiliations:** ^1^Department of Morphology, Surgery and Experimental Medicine and LTTA Centre, University of Ferrara, Via Fossato di Mortara 70, 44121 Ferrara, Italy; ^2^Department of Medical Science, Section of Internal and Cardiopulmonary Medicine, University of Ferrara, Via Aldo Moro 8, 44124 Cona, Ferrara, Italy; ^3^Department of Biomedical and Specialist Surgical Sciences, Section of Medical Biochemistry, Molecular Biology and Genetics, University of Ferrara, Via Luigi Borsari 46, 44121 Ferrara, Italy; ^4^Gerontology and Geriatric Research Laboratory, IRCCS Casa Sollievo della Sofferenza, San Giovanni Rotondo, Viale Cappuccini 1, 71013 Foggia, Italy; ^5^Department of Medical Sciences, University of Ferrara, Via Luigi Borsari 46, 44121 Ferrara, Italy; ^6^Plants for Human Health Institute, Department of Animal Sciences, NC Research Campus, North Carolina State University, 600 Laureate Way, Kannapolis, NC 28081, USA; ^7^Department of Life Sciences and Biotechnology, University of Ferrara, Via Luigi Borsari 46, 44121 Ferrara, Italy

## Abstract

**Background:**

Blood-based parameters reflecting systemic abnormalities associated with typical brain physiopathological hallmarks could be a satisfactory answer to the need of less costly/intrusive and widely available biomarkers for late onset Alzheimer's disease (LOAD). Cumulating evidence from ourselves and others suggests that systemic oxidative stress (OxS) is precociously associated with LOAD. On this basis, we aimed to identify a combination of markers of redox status that could aid the diagnosis of LOAD.

**Methods:**

We reexamined and crossed previous data on 9 serum markers of OxS obtained in a cohort including *n* = 84 controls and *n* = 90 LOAD patients by multivariate logistic regression analyses.

**Results:**

A multimarker panel was identified that included significantly increased (hydroperoxides and uric acid) and decreased (thiols, residual antioxidant power, and arylesterase activity) markers. The multivariate model yielded an area under receiver-operating characteristic curve (AUC) of 0.808 for the discrimination between controls and LOAD patients, with specificity and sensitivity of 64% and 79%, respectively.

**Conclusions:**

This study identified a panel of serum markers that distinguish individuals with LOAD from cognitively healthy control subjects. Replication studies on a larger independent cohort are required to confirm and extend our data.

## 1. Introduction

Dementia is one of the major causes of disability among elderly people, with more than one hundred million people worldwide estimated to suffer from this syndrome in 2050 [1]. Alzheimer's disease, especially the late onset subtype (LOAD), is the most frequent form of this syndrome, accounting for more than 70% of affected people [1].

Despite intense research, the current knowledge on LOAD pathogenesis is still incomplete [2]. Moreover, the most commonly held hypothesis that points to the accumulation of amyloid beta (A*β*) in the brain as the primary event has been questioned [3]. One of the main reasons accounting for these partially unsuccessful efforts relies on the multifaceted and multifactorial nature of the disease. This form of dementia is currently diagnosed through combination of costly/time-consuming imaging tests, psychological evaluation, or invasive A*β* and Tau determination in cerebrospinal fluid (CSF); the conclusive diagnosis still requires histopathological examination of brain tissue [4]. Although showing high analytical performance, CSF biomarkers have not yet achieved the widespread approval and availability necessary in clinical practice. One of the central concept in the definition of an ideal biomarker is that it should be “noninvasive, easily translatable to routine clinical testing or eventually high-throughput population screening, and expedient serial monitoring” [5]. Blood (serum/plasma) candidate biomarkers, especially when the detection methods are nonexpensive and easy to perform, potentially fit this definition. More difficult is to fulfill to the other key prerequisite for a biomarker which should directly reflect disease pathophysiology and be informative of the disease process, even in the preclinical phase.

As suggested elsewhere [6], peripheral biomarkers could reflect central nervous system (CNS) pathology only if at least one of the following hypotheses is true: (1) systemic processes drive brain dysfunction; (2) brain pathological changes drive systemic manifestations; and (3) a pathophysiological process occurs and evolves in parallel in CNS and periphery. Abundant evidence from our group and from others suggests that oxidative stress (OxS) is a good candidate for satisfying the last two hypotheses [7–11]. Fingerprints of OxS, caused by a derangement of a preexisting balance between oxidant and antioxidant species, have been found both in brain and in periphery of patients with the preclinical (i.e., mild cognitive impairment—MCI) or clinical stage of the disease [7, 10]. Evidence from animal and in vitro studies points to a direct role of the main biological oxidants (i.e., reactive oxygen species—ROS) in Alzheimer's disease [2, 12, 13].

Previous findings from our group [9–11, 14–16] have shown significant association between serum markers of redox status (ranging from oxidative damage by-products to single or collective parameters of antioxidant capacity) and MCI/LOAD diagnosis. Nevertheless, none of the tested markers (*n* = 9) discriminated with high clinical accuracy (i.e., >60%) healthy subjects from patients. These results were not surprising because of the heterogeneity of disease and the lack of a single parameter able to assess OxS. Owing these premises, in this study, we evaluated whether the combination of the different serum parameters of the redox state allows us to reach acceptable clinical sensitivity/specificity for the diagnosis of LOAD.

## 2. Materials and Methods

### 2.1. Subjects

Two hundred sixty-three elderly Caucasian outpatients (≥65 years) referring to the Internal Medicine (University of Ferrara, Italy) or to the Geriatric Unit of the IRCCS “Casa Sollievo della Sofferenza” (San Giovanni Rotondo, Italy). The research protocol was carried out according to the Declaration of Helsinki (World Medical Association, http://www.wma.net) and the European Guidelines for Good Clinical Practice (European Medicines Agency, http://www.ema.europa.eu). The study was approved by Local Ethics Committee of the involved institutions (Province of Ferrara, Italy, and Casa Sollievo della Sofferenza, San Giovanni Rotondo, Italy). The research protocol did not modify the routine clinical/diagnostic protocols implemented for the diagnosis of cognitive impairment/dementia in the memory clinic nor conditioned any decision about the treatments of the enrolled individuals. All participants (and/or their guardian/relative if demented) were informed about the research project and signed an informed consent.

Subjects with diagnosis of severe liver or kidney disease, severe chronic obstructive pulmonary, severe congestive heart failure, and cancer or taking nonsteroidal anti-inflammatory drug (NSAIDS), antibiotics, or steroids were excluded from the study. Personal data and medical history were collected by trained personnel from eligible patients and/or caregivers. General and neuropsychological examinations were carried as previously described [11]. Clinical chemistry analyses were routinely performed to exclude causes of secondary cognitive impairment. Trained geriatricians made the diagnosis of dementia as described elsewhere [11].

One hundred seventy-four subjects were finally considered in this study since they had complete demographic and health status information besides all the 9 OxS markers examined.

The sample included the following:

- 90 individuals with mild-moderate LOAD according to the National Institute of Neurological Disorders and Stroke—Alzheimer's Disease and Related Disorders Association criteria (MMSE range: 18–23) (CDR: 1-2). Moreover, these outpatients underwent brain computer tomography (by Siemens Somaton HQ) to support the clinical diagnosis and to evaluate other brain pathologies associated with cognitive impairment (e.g., cerebrovascular disease, metastasis, and normal pressure hydrocephalus).
- 84 control subjects (controls—C) with normal cognitive performance (MMSE range: 26–29).

### 2.2. Assays of Biochemical Parameters

Peripheral blood samples were collected by venipuncture into Vacutainer tubes without anticoagulant after an overnight fast. After 30 minutes of incubation at room temperature, the blood samples were centrifuged at 4.650 ×g for 20 min and sera were collected and stored in single-use aliquots at −80°C until analysis. With the exception of homocysteine, the assays were performed by using UV-VIS spectrophotometry in a 96-well plate format (Tecan Infinite M200 from Tecan Group Ltd., Switzerland).

- Hydroperoxides were assessed by colorimetric assay based on the reaction between these lipid peroxidation by-products and N,N-diethyl-para-phenylendiamine as we previously described in detail [11, 17]. Briefly, 20 *μ*L of serum or standard (H_2_O_2_) was added to a solution containing 1960 *μ*L of acetate buffer (pH 4.8) and 20 *μ*L of chromogen (0.0028 mol/L). The 505 nm absorbance of this solution was detected at time 0 and after 6 minutes of incubation at 37°C. Results were expressed as Carr units (CU) where 1 CU corresponds to 0.023 mmol/L of H_2_O_2_. The intra-assay coefficient of variation (CV) was 2.5%, whereas the interassay CV was 3.5%.
- Uric acid (*μ*mol/L) was determined by the direct enzymatic method [18] in which uric acid was oxidized by uricase coupled with peroxidase, and the results were measured colorimetrically. The intra-assay CV was 2.5%, whereas the interassay CV was 5.3%.
- Residual antioxidant power (RAP) was assayed in serum according to the description by Benzie and Strain [19] with modifications. The original method, universally known as FRAP (ferric reduction antioxidant power), measures the ability of water- and fat-soluble antioxidants contained in serum (e.g., uric acid, ascorbic acid, *α*-tocopherol, bilirubin, and vitamin A) to reduce ferric tripyridyltriazine (Fe^3+^-TPTZ) to the chromogenic ferrous form (Fe^2+^-TPTZ). Briefly, 300 mmol/L of acetate buffer, pH 3.6, 10 mmol/L TPTZ, and 20 mmol/L FeCl_3_ were mixed in the ratio 10 : 1 : 1 to give the working solution. 970 *μ*L of this solution was added to 30 *μ*L of serum or standard (ascorbic acid or FeSO_4_). The 593 nm absorbance of this solution was detected at 0 time and after 6 minutes of incubation at room temperature. Uric acid is the major contributor of total reducing power of serum. The constant stoichiometric factor of this test (1 *μ*mol/L = 2 FRAP units) allows the determination of a residual antioxidant power, by subtracting the contribution of uric acid from the total antioxidant power values. Therefore, RAP is a parameter affording a further index of antioxidant status in uric acid-rich fluids such as serum [19, 20]. Results were expressed as FRAP units, where 1 FRAP corresponds to 100 *μ*mol/L of Fe^3+^ reduced to Fe^2+^. The intra-assay CV was 3.9%, whereas the interassay CV was 9.9%.
- Thiols (TH) (*μ*mol/L) was spectrophotometrically determined by the colorimetric dithionitrobenzoic acid- (DTNB-) based assay according to Hu's method, and L-cysteine was employed as standard [21]. Ten *μ*L of serum was added to 990 *μ*L of working solution containing 0.2 phosphate buffer (pH 8.0) and 0.25 mmol/L of DTNB. Following 5 minutes of incubation at room temperature, absorption at 405 nm was measured. The intra-assay CV was 6.5%, whereas the interassay CV was 8.5%.
- Advanced oxidation protein products' (AOPP) determination was based on spectrophotometric detection according to Capeillère-Blandin et al. [18]. Two hundred *μ*L of serum diluted 1 : 5 in phosphate-buffered saline and 20 *μ*L of acetic acid were placed in each well. For the standard, 10 *μ*L of 1.16 mmol/L of potassium iodide and 20 *μ*L of acetic acid were added to 200 *μ*L of chloramine-T solution (0 to 100 *μ*mol/L). The absorbance of the reaction mixture was immediately read at 340 nm. Concentrations of AOPP determined in reference to the calibration were expressed in *μ*mol/L. The intra-assay CV was 5.1%, whereas the interassay CV was 9.5%.
- Arylesterase enzymatic activity of paraoxonase-1 (PON-1) was assayed by measuring initial hydrolysis rates of phenylacetate as described in detail in previous reports [14, 16, 22]. The arylesterase activity was measured by adding 10 *μ*L of serum, diluted 24 times, to 240 *μ*L of reaction mixture composed by 1 mmol/L phenylacetate and 0.9 mmol/L CaCl_2_ dissolved in 9 mmol/L Tris-HCl, pH 8. One unit of arylesterase activity accounts for 1 *μ*mol of phenol produced in a minute under the standardized conditions of the assay. The intra-assay CV was 3.8%, whereas the interassay CV was 9.7%.
- Paraoxonase activity assay was carried out by assessing the rate of formation of para-nitrophenol, which is derived by catalyzed hydrolysis of paraoxon [14]. This activity was assayed by continuous monitoring of the increase in the absorbance at 412 nm caused by 4-nitrophenol formation after addition of 5 *μ*L of serum in 245 *μ*L of reaction mixture consisting 1.5 mmol/L paraoxon, 0.9 mol/L NaCl, and 2 mmol/L CaCl_2_ dissolved in 10 mmol/L Tris-HCl, pH 8. A molar extinction coefficient of 18 × 10^3^ L^−1^ mol^−1^ cm^−1^ was used for the calculation of enzyme activity, expressed in units per liter. One unit of paraoxonase activity is defined as 1 *μ*mol of 4-nitrophenol formed per minute under the given conditions. The intra-assay CV was 4.9%, whereas the interassay CV was 6.9%.
- The homocysteine level was assessed by the Liquid Stable (LS) 2-Part Homocysteine Reagent (Axis-Shield Diagnostics Ltd., UK) using the Roche COBAS Integra 800 Chemistry Analyzer, according to the manufacturer's instructions. The concentrations of homocysteine, determined in reference to the calibration curve, were expressed in *μ*mol/L. The intra-assay CV was 1.5%, whereas the interassay CV was 2.6%.
- Total ferroxidase (total FeOx) activity was measured in serum samples according to Erel's method [23] with some minor modifications. This assay measures the rate of oxidation of Fe^2+^ to Fe^3+^ (by using 3-(2-pyridyl)-5,6-bis-[2-(5-furylsulfonic acid)]-1,2,4-triazine as chromogen) catalyzed by ceruloplasmin and other soluble factors. Briefly, 5 *μ*L of sample was added to 195 *μ*L of acetate buffer (0.45 mol/L, pH = 5.8). After 1 min incubation at 37°C, 43 *μ*L of 370 mmol/L Fe(NH_4_)_2_SO_4_ was added and the resulting mixture was incubated for a further 4 min at 37°C. At the end of the incubation, 20 *μ*L of chromogen was added. The rate of formation of colored complex (formed by the chromogen and ferrous ions) was recorded at 600 nm. The difference in the ferrous ion concentration before and after the enzymatic reaction indicated the amount of oxidized ferrous ion. The amount of enzyme that converted 1 *μ*mol of substrate into product per minute in one liter of sample was defined as 1 U/L. The intra-assay CV was 2.5%, whereas the interassay CV was 9.0%.

### 2.3. Statistical Analysis

Continuous variables were first analyzed for normal distribution using Kolmogorov-Smirnov and Shapiro-Wilkinson tests. Comparisons between groups were performed using the *t*-test and Mann–Whitney *U* test for normally and nonnormally distributed variables, respectively. Chi-squared tests were used to compare differences in categorical variables.

The nine OxS parameters (hydroperoxides, AOPP, RAP, thiols, uric acid, homocysteine, paraoxonase, arylesterase, and total FeOx) were evaluated as possible LOAD predictors, using simple logistic regression models. Parameters were dichotomized in order to detect the range of “risk values”; the cut-off used for categorization was the value corresponding to the best compromise between sensibility and specificity.

Multivariate logistic regression analysis was used to estimate the net effect of each (significant at univariate analysis) risk factor. The forward selection procedure was used starting from the parameter with the best discriminatory power (highest AUC) and adding on any other significant parameter until the improvement between consecutive models was minimal (AUC improvement < 0.01). Collinearity among the parameters considered was checked before performing the multivariable analysis.

Successively, a score indicating the risk gravity was created using the regression coefficients of the multivariate model. The number of points assigned to each predictor was set equal to its regression coefficient divided by the smallest one; the quotient was rounded to the nearest integer number, and the score for each patient was calculated by summing up the points related its risk factors. Again, the ability of the score to discriminate between control and LOAD was calculated using the area under receiver operating characteristic (ROC) curve.

Finally, a simpler score was obtained summing up the number of risk factors, that is, giving the same weight (score point: 1) to each of them. The given parameter was considered as a risk factor when its level was higher (in case of positive association with LOAD risk) or lower (if negatively associated) than the cut-off. Thus, score 1 indicates that the subjects present only one risk factor (e.g., arylesterase level lower than its cut-off or hydroperoxide level higher than its cut-off); score 2 = two risk factors (e.g., arylesterase level lower and hydroperoxide level higher than their respective cut-offs); score 3 = three risk factors; score 4 = four risk factors; score 5 = five (all) risk factors.

A *p* value < 0.05 was considered statistically significant. All analyses were run using Stata 13.

## 3. Results

Table 1 provides a summary of the sociodemographic, lifestyle, health status, and serum parameters of OxS (hydroperoxides, AOPP, RAP, thiols, uric acid, homocysteine, paraoxonase, arylesterase, and total FeOx) of the study sample (*n* = 174). Females were slightly more prevalent in controls compared to LOAD (*p* < 0.05). Controls were younger (*p* < 0.01) and displayed a lower prevalence of hypertension and higher prevalence of stroke, compared to LOAD (*p* < 0.001); no significant differences emerged as regards CVD and diabetes. As expected, LOAD had a lower MMSE score and education level (*p* < 0.001 for both comparison).

RAP and arylesterase were lower in LOAD (*p* < 0.001) compared to controls. Thiols also showed a similar trend (*p* < 0.05), whereas uric acid and homocysteine levels were both increased in LOAD compared to controls (*p* < 0.01). Finally, no significant differences were observed for AOPP, hydroperoxides, paraoxonase, and total FeOx.

The ability of OxS parameters to discriminate LOAD from controls was initially checked by logistic regression analysis (Table 2). From these preliminary tests, five parameters emerged as significantly associated with LOAD diagnosis by using the best cut-off values. Considering the AUC values, the best discriminatory parameters were (in order of magnitude) uric acid, thiols, arylesterase, RAP, and hydroperoxides. Of note, none of the above parameters reached the described acceptability criteria of 0.70.

**Figure 1:**
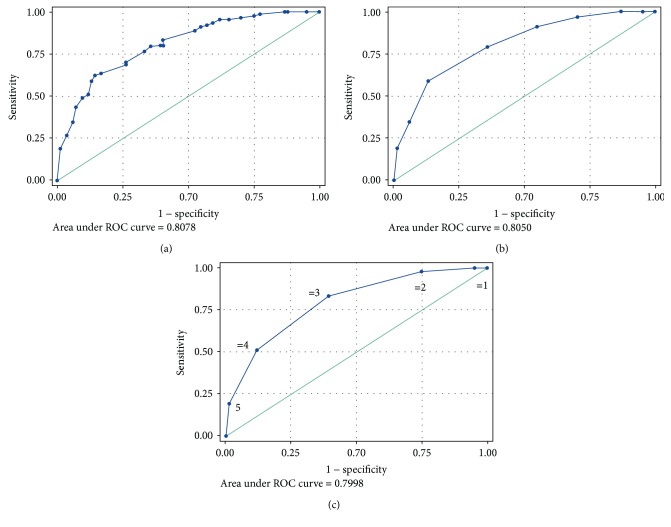
Receiver operating characteristic (ROC) curves for multimarker panel (including hydroperoxides, uric acid, residual antioxidant power, thiols, and arylesterase) for the diagnosis of late onset Alzheimer's disease. (a) Multivariate logistic model: specificity/sensitivity = 64%/79%; (b) score model: specificity/sensitivity = 61%/83%; (c) number of risk factor model; specificity/sensitivity = 61%/83%. Chart C: value “1” means that the subject present only one risk factor (e.g., thiols lower than its cut-off); “2”, subjects with two risk factors (e.g., thiols and arylesterase lower than their respective cut-offs); “3”, three risk factors and so on. See Materials and Methods section or the description of the models used for ROC calculation.

To improve the prediction power for LOAD, we performed a multivariate logistic regression model including the parameters associated in univariate analysis. By adding one by one the five significant parameters (from strongest to weakest predictor), the AUC for LOAD increased to a final value of 0.808 (online resource: Supplementary Table 1 and Figure 1(a)). Using ROC analysis, we identified the best compromise between specificity and sensitivity for the diagnosis of LOAD (64.3% and 79%, respectively, accuracy = 71.8%; Figure 1(a)), which was calculated by considering as positive subjects with >0.5 probability.

The ability to discriminate LOAD from controls was also investigated by using two other different models. We first created a model (maximum score = 8), calculated by using scores as displayed in online resource: Supplementary Table 2. Using ROC analysis, we calculated an optimal cut-off of ≥5 for identifying LOAD patients, with specificity and sensitivity of 64% and 79%, respectively (Figure 1(b)). AUCs were very similar to that calculated with the first multivariate analysis (AUC = 0.805). Finally, the ROC curve obtained by considering the simple number of risk factors did not significantly improve the statistical outcome. The AUC for LOAD diagnosis was 0.79, and the best specificity and sensitivity (61% and 83%) were obtained with a cut-off of ≥3 risk factors (Figure 1(c)).

The significant difference in age between controls and LOAD along with the (although weak) correlation between age and the five serum markers (online resource: Supplementary Table 3) prompted us to evaluate the potential effect of this variable on the prediction power of the multimarker panel. We found that for LOAD diagnosis, adding the dichotomous variable “age > 70 years” (70 was the median age for the whole sample) to the multivariate logistic model resulted in (1) no loss of significance of any serum markers and (2) increase of AUC from 0.807 to 0.899 (data not shown).

## 4. Discussion

The studies we published in the last few years witness our commitment in seeking reliable peripheral markers for the diagnosis of LOAD and other dementias. We targeted serum as best biological fluid because of its high accessibility and suitability for repeated sampling and analyses. This is an important point to be addressed in biomarker field study. Indeed, a biomarker to be defined as useful in a clinical setting should not solely satisfy the essential analytical-clinical requirements, but it also should be easy to measure and inexpensive. Pursuing this path, we found that a group of serum markers with the aforementioned features, commonly employed to evaluate systemic redox balance, was altered in cases compared to controls [9–11, 14–16]. In this observational study, we gathered together and crossed the data generated in the previous studies, with the aim to find a combination of serum markers with high diagnostic accuracy for LOAD. The two multimarker panels generated from logistic and ROC analysis discriminated patients with LOAD from cognitively normal controls with AUC of approximately 80%, and a specificity/sensitivity of 64.3/78.9%. The combinations of the five best markers led to an average 20% increase of diagnostic accuracy compared to that yielded by single markers. As observed in other studies [24], accuracy was greatly improved by incorporating also age into the analysis. Intriguingly, comparable statistical outcomes were obtained by using a simpler model that considered just the number but not the type of “positive” markers.

The multimarker panel we tested includes indexes of oxidative damage (hydroperoxides) and of antioxidant defense, ranging from nonenzymatic (uric acid, thiols, and those contributing to residual antioxidant power) to enzymatic (arylesterase). Changes in serum concentration/activity of these different factors reflect modification in systemic redox balance. As classically defined, the two sides of this physiological balance are occupied by two counteracting chemical/biochemical species, oxidants (ROS, reactive oxygen species, are the most abundant), and antioxidants. Oxidative challenge to biomolecules, an event often underlying the onset of several diseases, is “the consequence of the failure to maintain the physiological redox steady state, which is the self-correcting physiological response to different challenges” [25]. Hydroperoxides, prominent nonradical intermediates of the oxidative modification of unsaturated fatty acids in cell membranes and lipoproteins, are one of the mostly measured by-products of oxidative processes triggered by ROS [25–27]. Hydroperoxides, together with malondialdehyde (MDA), F2-isoprostanes, and 4-hydroxynonenal (4-HNE), have been repeatedly associated with LOAD and also MCI risk [9, 11, 28–31].

As opposed to hydroperoxide increase, also a decrease in antioxidant levels could be an index of OxS. The lower levels of residual antioxidant power in LOAD reflect lower levels of vitamins E and C or *β*-carotene, which mostly contribute to this serum parameter [11, 19] and have been shown to decrease in patients with dementia [32–34]. The other two nonenzymatic antioxidants included in the panel, thiols (mostly sulfhydryls of cysteines in albumin) and uric acid, are regarded as the most abundant endogen antioxidants in the plasma [19, 35]. It is fair to underscore that the impact of uric acid in the global redox state is still debated. In one hand, this end-product of purine catabolism is a chelator of transitional metal ions, which in the free form constitute a major prooxidant factor. In the other hand, xanthine oxidase (which is involved in the synthesis of uric acid), when upregulated, generates superoxide radical (highly reactive ROS); moreover, hyperuricemia is a well-documented risk factor of CVD [35]. This paradoxical role might account for the significant association between higher levels of uric acid and MCI or LOAD we observed, as and for the alternate results reported in literature [36].

Arylesterase activity of PON-1 was included into the multimarker panel because it was a strong predictor of LOAD [14]. Albeit PON-1 paraoxonase activity has been related to the protection from toxic metabolites of organophosphorus pesticides (and more influenced by genetic polymorphisms [37]), PON-1 arylesterase activity has been found to be strictly associated with several diseases besides LOAD, such as multiple sclerosis, vascular dementia, and CVD [15, 38, 39]. This epidemiologically ascertained association might be due to the in vitro and in vivo ability of the enzyme to contribute to antioxidant and anti-inflammatory function of HDL particles, its major carrier [39].

A contextualization of our findings in the current literature is complicated since our approach, consisting in the evaluation of the global discriminatory potential of a number of different OxS markers, is unprecedented, at least in Alzheimer's disease field. On the contrary, a large number of studies in recent years have been focused on panels of multiple blood proteins able to predict LOAD. The pioneering study in this research was made by Ray et al. who measured the relative concentrations of 120 cell signaling proteins (ranging from growth factors to cytokines and other proteins involved in immune response) in plasma [40]. The authors observed that the concentrations of 18 plasma proteins were able to classify AD and, most importantly, MCI which subsequently converted to AD, with an accuracy close to 90%. Unfortunately, the high diagnostic accuracy of this biomarker signature has not been confirmed by subsequent replicating studies on independent cohorts [41–43], also due to the use of different detection assays [43]. Since Ray et al.'s first report, the focus on blood-based protein biomarkers for LOAD has grown exponentially. Unfortunately, although protein patterns achieved high specificity/sensitivity for the diagnosis of the disease, protein patterns were rarely superimposable to each other [24, 43, 44].

In spite of the lack of definitive confirmation of the clinical usefulness of protein signatures, it is undeniable that the path toward the discovery of effective biomarkers for LOAD has been already traced. We firmly agree with the concept that biomarkers should be “combinational” because this is the only way they can picture the high complexity and heterogeneity of disease pathogenesis/pathophysiology [44, 45]. Our results, along with others, clearly suggest that the putative multiple marker panel might combine blood proteins (revealed by multiplex immunoassays or classical proteomic approach) with other low-weight substances or enzymes dealing with OxS. An unbalanced redox state could be one of the aberrant processes, along with copper/iron dyshomeostasis, endothelial dysfunction, and low-grade inflammation, coexisting in both brain and periphery since the preclinical stage of the disease. The early determination of the “weight” of a given pathophysiological component by an easy, fast, and cheap assay may help in identifying patients subpopulations that might benefit from certain treatments (antioxidants if OxS is elevated), allowing patient stratification in clinical trials.

We are aware about important limitations of our study that must be finally acknowledged. First, the small size of the sample may affect the reliability and clinical significance of the results. Second, the cross sectional design of the study does not allow us to draw any conclusion on the cause-effect relationship between disease and markers. Third, it might be argued that some of the markers included in our panel (particularly arylesterase and uric acid) are not universally accepted as specific indicators of OxS. We are aware that the five markers do not exclusively reflect redox status but also other physiopathological conditions (e.g., inflammation and dyslipidemia) and metabolic disturbances characterizing this multifactorial disease. Fourth, we cannot exclude any other biases due to the still unstandardized methods we used for marker determination and from differences in comorbidities and gender between controls and LOAD patients. Thus, replication studies in independent cohorts are warranted to confirm our data and translate them into clinic.

## 5. Conclusion

At present, the diagnosis of LOAD requires a combination of complex, labor-consuming, and expensive clinical examinations. Our results, showing a panel of five serum markers of redox status discriminated between LOAD patients and controls with “fair” sensitivity and specificity, suggest that blood could be a potential source of easy and time-sparing biomarkers for the diagnosis of this disease. However, the potential limitations of the study (cross sectional design, small size of the sample) strongly suggest the need to replicate our results in an independent cohort.

## Figures and Tables

**Table 1:** Principal characteristics of the sample according to diagnosis.

	Controls (*n* = 84)	Load (*n* = 90)
Age (years)^∗^	69 ± 9	77 ± 6
Sex (females, %)^∗^	89	74
MMSE score^∗^	28 (25–29)	21 (18–24)
Education (yrs)^∗^	8 (5–13)	5 (3–6)
Current smokers (%)	12	19
Hypertension (%)^∗^	35	67
Diabetes (%)	11	15
CVD (%)	12	13
Stroke (%)^∗^	4	0
Serum parameters		
AOPP (*μ*mol/L)	73 (62–92)	69 (61–78)
Hydroperoxides (CU)	295 (201–374)	331 (205–396)
RAP (FRAP units)^∗^	223 (131–315)	138 (90–227)
Thiols (*μ*mol/L)^∗^	213 (115–260)	174 (111–245)
Uric acid (*μ*mol/L)^∗^	300 ± 96	357 ± 95
Homocysteine (*μ*mol/L)^∗^	13 (9–18)	16 (11–22)
Paraoxonase (U/L)	84 (48–163)	92 (48–154)
Arylesterase (kU/L)^∗^	110 ± 32	86 ± 25
Total FeOx (U/L)	524 (457–602)	558 (477–631)

Mean ± SD for normally distributed variables; median (interquartile range) for not normally distributed variables; percentage for discrete variables. Abbreviations: LOAD: late onset Alzheimer's disease; CVD: cardiovascular disease; AOPP, advanced oxidation protein products; total FeOx, total ferroxidase; CU, Carr units (1 CU = 0.023 mmol/L of H_2_O_2_); FRAP units, ferric reduction antioxidant power (1 FRAP unit = 100 *μ*mol/L of Fe^3+^ reduced to Fe^2+^); RAP, residual antioxidant power. ^∗^ *p* < 0.05*t*-test, Mann–Whitney *U* test, or chi-squared test (prevalence).

**Table 2:** Cut-off values, odds ratios, and AUCs for the diagnosis of LOAD calculated for each single serum parameter.

	Cut-off^∗^	OR (*p*)	AUC
AOPP	70 *μ*mol/L	1.66 (*p* = 0.075)	0.563
Hydroperoxides	330 CU	1.72 (*p* = 0.035)	0.566
RAP	190 FRAP units	3.18 (*p* < 0.001)	0.640
Thiols	190 *μ*mol/L	3.16 (*p* < 0.001)	0.640
Uric acid	279 *μ*mol/L	4.14 (*p* < 0.001)	0.653
Homocysteine	14 *μ*mol/L	2.17 (*p* = 0.013)	0.595
Paraoxonase	90 U/L	1.22 (*p* = 0.375)	0.525
Arylesterase	94 kU/L	2.89 (*p* < 0.001)	0.629
Total FeOx	510 U/L	1.56 (*p* = 0.097)	0.554

^∗^Cut-off points corresponding to the best compromise between specificity and sensitivity. Abbreviations: OR, odds ratio; LOAD: late onset Alzheimer's disease; CVD: cardiovascular disease; AOPP, advanced oxidation protein products; total FeOx, total ferroxidase; RAP, residual antioxidant power; CU, Carr units (1 CU = 0.023 mmol/L of H_2_O_2_); FRAP units, ferric reduction antioxidant power (1 FRAP unit = 100 *μ*mol/L of Fe^3+^ reduced to Fe^2+^).

## Data Availability

The data are available on request and will be provided by Professor Carlo Cervellati (email: crvcrl@unife.it) or Professor Giovanni Zuliani (email: giovanni.zuliani@unife.it).

## Supplementary Materials

Supplementary Materials Supplementary Table 1: the table shows the results of multivariate logistic models for LOAD diagnosis including different combinations of uric acid, thiols, residual antioxidant power, arylesterase, and hydroperoxides. Supplementary Table 2: the table shows the regression coefficients for the association between serum makers and LOAD, and calculation of the scores used for ROC curve analysis. Supplementary Table 3: the table shows the simple correlation coefficients (*r*) between age and serum parameters included in multimarker panel.
