# Supplementary material for: Testing a Combination of Markers of Systemic Redox Status as a Possible Tool for the Diagnosis of Late Onset Alzheimer's Disease
Source: Dis Markers. 2018 Sep 9;2018:2576026. doi: 10.1155/2018/2576026 (PMC6151249; doi:10.1155/2018/2576026)
Supplement: Supplementary Materials — Supplementary Table 1: the table shows the results of multivariate logistic models for LOAD diagnosis including different combinations of uric acid, thiols, residual antioxidant power, arylesterase, and hydroperoxides. Supplementary Table 2: the table shows the regression coefficients for the association between serum makers and LOAD, and calculation of the scores used for ROC curve analysis. Supplementary Table 3: the table shows the simple correlation coefficients (r) between age and serum parameters included in multimarker panel. [file 2576026.f1.doc]

| **Supplementary Table 1**: Multivariate logistic models for LOAD diagnosis including different combinations of uric acid, thiols, residual antioxidant power, arylesterase and hydroperoxides | | | | | |
| --- | --- | --- | --- | --- | --- |
| **Logistic models** | **Predicting variables** |  | **OR** | **AUC** | **p^*^** |
| **1** | **Uric acid** |  | 4.25 | 0.656 | <0.001 |
| **2** | **-Uric acid**  **-Thiols** |  | 2.39 | 0.742 | 0.019 |
| **3** | **-Uric acid**  **-Thiols**  **-RAP** |  | 3.18 | 0.774 | 0.008 |
| **4** | **Uric acid**  **-Thiols**  **-RAP**  **-Arylesterase** |  | 4.16 | 0.800 | <0.001 |
| **5** | **-Uric acid**  **-Thiols**  **-RAP**  **-Arylesterase**  **-Hydroproxides** |  | 2.04 | 0.808 | 0.05 |
| ^*^calculated for multivariate model  Cut-off points corresponding to the best compromise between specificity and sensitivity  Abbreviation: OR, odds ratio; LOAD: late onset Alzheimer’s disease; RAP, residual antioxidant power | | | | | |

| **Supplementary Table 2:** Regression coefficients for the association between serum makers and LOAD, and calculation of the scores used for ROC curve analysis | | | | |
| --- | --- | --- | --- | --- |
| **Serum parameters** |  | **Diagnosis of LOAD** | | |
|  |  | **Regression coefficient** | **Calculated score^*^** | **Final score** |
| **Hydroperoxides** |  | 0.711 | 1 | 1 |
| **RAP** |  | 0.870 | 1.22 | 1 |
| **Residual antioxidant power** |  | 0.249 | 1.61 | 2 |
| **Arylesterase** |  | 1.426 | 2.00 | 2 |
| **Hydroperoxides** |  | 1.448 | 2.04 | 2 |
| ^*^calculated by dividing the regression coefficient by the smallest one (first line)  Abbreviation: LOAD: late onset Alzheimer’s disease; RAP, residual antioxidant power | | | | |

| **Supplementary Table 3:** Simple correlation coefficients (r) between age and serum parameters included in multi-markers panel . | | | | | |
| --- | --- | --- | --- | --- | --- |
|  | **Hydroperoxides**  **r (p)** | **Residual antioxidant power**  **r (p)** | **Thiols**  **r (p)** | **Uric acid**  **r (p)** | **Arylestrase**  **r (p)** |
|  | -0.207 (0.006)^*^ | 0.003 (0.968) ^*^ | -0.236 (0.002) ^*^ | 0.296 (0.041)# | -0.153 (0.044) # |
| ^*^Pearson’s coefficient; ^#^Spearman’s correlation coefficient | | | | | |
